# Quantitative Systems Pharmacology and Biased Agonism at Opioid Receptors: A Potential Avenue for Improved Analgesics

**DOI:** 10.3390/ijms23095114

**Published:** 2022-05-04

**Authors:** Andrea Bedini, Elisabetta Cuna, Monica Baiula, Santi Spampinato

**Affiliations:** Department of Pharmacy and Biotechnology (FaBiT), University of Bologna, 40126 Bologna, Italy; elisabetta.cuna2@unibo.it (E.C.); monica.baiula@unibo.it (M.B.); santi.spampinato@unibo.it (S.S.)

**Keywords:** opioid receptors, biased agonism, multidimensional signaling network, improved analgesics, Quantitative Systems Pharmacology

## Abstract

Chronic pain is debilitating and represents a significant burden in terms of personal and socio-economic costs. Although opioid analgesics are widely used in chronic pain treatment, many patients report inadequate pain relief or relevant adverse effects, highlighting the need to develop analgesics with improved efficacy/safety. Multiple evidence suggests that G protein-dependent signaling triggers opioid-induced antinociception, whereas arrestin-mediated pathways are credited with modulating different opioid adverse effects, thus spurring extensive research for G protein-biased opioid agonists as analgesic candidates with improved pharmacology. Despite the increasing expectations of functional selectivity, translating G protein-biased opioid agonists into improved therapeutics is far from being fully achieved, due to the complex, multidimensional pharmacology of opioid receptors. The multifaceted network of signaling events and molecular processes underlying therapeutic and adverse effects induced by opioids is more complex than the mere dichotomy between G protein and arrestin and requires more comprehensive, integrated, network-centric approaches to be fully dissected. Quantitative Systems Pharmacology (QSP) models employing multidimensional assays associated with computational tools able to analyze large datasets may provide an intriguing approach to go beyond the greater complexity of opioid receptor pharmacology and the current limitations entailing the development of biased opioid agonists as improved analgesics.

## 1. Adequate Management of Chronic Pain: Still a Relevant Unmet Need

Chronic pain is a debilitating condition affecting about 20% of the European population and deeply impacting the patients’ quality of life and ability to carry out professional and personal activities [1], often leading to secondary problems such as depression, sleeping disturbance, fatigue, and decreased cognitive function [2,3]. Furthermore, chronic pain poses a relevant socio-economic burden due to expenses for medications and therapeutic intervention and to patients’ loss of productivity (e.g., absenteeism, reduced work performance, long-term disability) [4]. It has been estimated that chronic pain costs about EU 200 billion/year in Europe and USD 150 billion/year in the US [5].

Chronic pain is more prevalent in women than men, increasing with age [6]. It has been reported that pain in women is more frequent, severe, and long-lasting as compared to men [7] and that there are relevant sex differences in neuronal and neuroimmune mediation of pain [8]. Moreover, females and males displayed different responses to analgesic drugs, thus, highlighting the dimorphic nature of pain and analgesia [9].

As regards the elderly, comorbidities and polypharmacy represent additional challenges in managing chronic pain states in this specific subset of patients [10]. Furthermore, it has been reported that elderly subjects are more susceptible to developing prolonged pain and are less responsive to different analgesic medications [11]. Gender differences in nociception and analgesia and concomitant age-related conditions, therefore, further expand the complexity of managing chronic pain effectively and safely.

Different classes of therapeutics are currently available to treat chronic pain, including non-steroidal anti-inflammatory drugs (NSAIDs), antidepressants, antiepileptics, and opioids; these latter, in particular, are still the mainstay in the treatment of moderate to severe pain, both acute and chronic, despite their relevant side effects and their abuse liability. Opioid analgesics (e.g., morphine, fentanyl, oxycodone) may indeed determine a significant effect on both pain perception and the emotional, cognitive, and behavioral responses to pain states [12]. However, their clinical use is limited by a plethora of adverse effects, including constipation, immune suppression, respiratory depression, tolerance, and abuse liability [13].

As a matter of fact, 60% of chronic pain patients treated with analgesics report inadequate pain relief or suffer from relevant adverse effects. Moreover, in the last decade, the misuse of prescription opioids has dramatically increased, with 4% of the adult population misusing opioids and more than 90 overdose deaths per day only in the US [14].

From all these observations, it clearly emerges the urgent unmet need for more effective and safer analgesics that may allow for adequate pain relief in chronic pain patients (including distinct subsets such as women or the elderly), with limited adverse effects and negligible abuse liability.

## 2. Biased Agonism at Opioid Receptors: A Possible Avenue for More Effective and Safer Therapeutics?

Biased agonism (also known as functional selectivity or ligand-directed signaling) is the ability of a ligand at a G protein-coupled receptor (GPCR) to activate distinct signaling pathways over others. This selective modulation of receptor activation is possible as GPCRs are dynamic entities existing in multiple conformations. Different agonists at the same receptor, therefore, may specifically stabilize one active conformation over another, thus, leading to the preferential activation of distinct subsets of signaling pathways [15,16]. Hence, since some intracellular transduction pathways may be mostly responsible for the therapeutic effects, whereas other signaling events may be mainly related to unwanted responses, biased agonism at GPCR has emerged as an intriguing opportunity to design tailor-made therapeutics [17].

The endogenous opioid system includes four receptors (mu-opioid or MOR; delta opioid or DOR; kappa opioid or KOR, nociceptin receptor or NOPr) and their endogenous peptide ligands (enkephalins, endorphins, endomorphines, dynorphins, nociceptins), each displaying distinct receptor affinity and selectivity [18]. From a therapeutic perspective, MOR is the most relevant opioid receptor as it is the main pharmacological target of opioid analgesics (e.g., morphine, fentanyl, oxycodone). 

Opioid receptors are class A GPCRs involved in various physiological and pathophysiological events, including pain, immune modulation, and emotional responses [18]. Once activated by an agonist, opioid receptors trigger Gα_i_-dependent inhibition of adenylyl cyclase, Gβγ-mediated activation of post-synaptic potassium channels, inhibition of pre-synaptic calcium channels, and activation of MAPKs (i.e., ERK1/2, p38MAPK, JNK). Prolonged stimulation of opioid receptors then leads to receptor phosphorylation and arrestin recruitment, that in turn modulates receptor internalization and desensitization as well as triggering arrestin-dependent signaling pathways [18]. Overall, opioid-mediated signal transduction is complex and multidimensional, thus, accounting for the wide variety of physiological and pathophysiological responses modulated by endogenous opioids as well as for therapeutic and adverse effects elicited by opioid drugs.

Agonists able to preferentially trigger G protein-mediated over arrestin-dependent intracellular signaling have been reported for opioid receptors [19], with G protein-biased agonists at MOR and KOR attracting the greatest interest in the frame of developing innovative therapeutics with improved analgesia and reduced side effects.

The idea of exploiting biased agonism at opioid receptors as an avenue for more effective and safer analgesics is hypothesis-driven: morphine administered to arrestin 3 knockout mice was shown to produce enhanced analgesia with reduced tolerance and fewer adverse events compared to wild-type mice [20,21]. Consistently, similar improved morphine potency with reduced tolerance was reported in mice and rats treated with siRNAs aimed to knock-down arrestin 3 expression [22,23]. On the other hand, numerous MOR agonists have been developed over the years in a global effort to improve opioid safety and tolerability, but all these structurally diverse opioids display morphine-like side effects (tolerance, nausea, vomiting, sedation, constipation, respiratory depression) [24]. Within a drug discovery paradigm mainly focused on identifying new receptor ligands with increasing affinity and specific characteristics at a single target, the hypothesis that MOR agonists preferentially activating G protein-dependent signaling, with a limited activation of arrestin-mediated pathways, may lead to an improved pharmacological profile seemed to be a highly promising strategy to overcome the classic adverse effects and limitations displayed by opioid analgesics. This has greatly spurred the quest for G protein-biased MOR agonists by many research groups and companies and progressively increased the expectations of functional selectivity at opioid receptors. Indeed, within the different approaches available to search for novel, safer, and less addictive opioid analgesics, biased agonism has received the most attention in recent years [25].

Nonetheless, despite the extensive research carried out on G protein biased MOR agonists and the expanding body of evidence that has accumulated, no G protein-biased MOR agonist has been approved by regulatory agencies as better than morphine; thus, weakening the whole concept of exploiting biased agonism at MOR as a new route to develop analgesics with an improved pharmacological profile [26].

Moreover, more recent literature data challenged the hypothesis of G protein bias at MOR as the molecular determinant of improved pharmacology.

Respiratory depression induced through MOR is at least partially mediated by receptor coupling to GIRK channels through the activation of Gβγ proteins [27]; furthermore, neurons in several regions of the brainstem respiratory network are hyperpolarized by activation of classical, arrestin-independent MOR signaling pathway [28]. Additionally, a recent study showed that the opioid side effect profile is not improved in a knock-in mouse model expressing phosphorylation deficient, G protein biased MOR (i.e., a MOR variant preferentially triggering G protein-dependent signaling regardless of the activating agonist) [29].

All these findings clearly demonstrate that G protein-dependent signaling contributes to at least some components of opioid-induced adverse effects on respiratory functions. On top of that, it should be considered that robust physiological evidence for arrestin signaling from MOR affecting respiratory function is absent [30], and that several laboratories have been unable to repeat the primary result of reduced morphine respiratory depression in arrestin knockout mice [31].

Explanations alternative to functional selectivity have been therefore proposed to account for the improved pharmacological profiles displayed by some MOR agonists initially reported as G protein biased, including low intrinsic efficacy at G protein [32]. G protein bias at MOR as an avenue for improved analgesics, therefore, is currently highly debated and controversial [33].

Nonetheless, functional selectivity at receptors other than MOR was connected to improved pharmacological profiles (e.g., kappa opioid receptor, 5HT1 receptor, beta-adrenergic receptor), and different endogenous opioid peptides were shown to favor particular signaling pathways at the three opioid receptors leading to biased signaling [34]. 

Hence, considering this multifaceted scenario, the potential utility of biased agonists at MOR cannot be completely ruled out yet. As a matter of fact, further studies are necessary to fully dissect the multidimensional complexity of MOR-mediated intracellular signaling and to connect the distinct signal transduction pathways to specific effects in vivo, to ultimately understand if, and to what extent, biased agonism at MOR may be exploited to develop more effective and safer analgesics.

KOR agonists, on the other hand, have attracted increasing interest in the last decade as alternatives to classic MOR analgesics, due to their reduced adverse effects on respiratory and gastrointestinal functions and to their low abuse potential [35]. Nonetheless, the clinical use of KOR agonists as pain killers in humans is strongly limited due to their relevant central adverse effects, including dysphoria, sedation, and motor incoordination [17].

Interestingly, it has been demonstrated that analgesic and anti-itch effects elicited by classic KOR agonists (e.g., U50,488, U69,593) are mediated through the activation of G protein-dependent signaling, while some of their relevant adverse effects (i.e., dysphoria, anhedonia, sedation, coordination impairment) occur following the induction of arrestin-mediated signaling events [26]. Thus, raising an increasing interest in developing G protein-biased KOR agonists as innovative analgesics with an improved efficacy/safety profile.

This hypothesis has been strongly corroborated by a large body of evidence that has accumulated in the last ten/fifteen years: arrestin 3-dependent induction of p38MAPK was reported to be responsible for inducing sedation, motor incoordination [36], centrally mediated dysphoria [37,38], potassium channel heterologous desensitization [39], neuropathy-induced astrocyte proliferation and related hyperalgesia [40]. 

Triazole 1.1 and RB-64, on the other hand, were reported as G protein biased KOR agonists inducing antinociceptive effects without impairing locomotion [41], and LOR17 was reported as a G protein biased KOR agonist eliciting anti-hypersensitivity effects in a mouse model of chemotherapy-induced neuropathic pain without determining significant sedation, impaired locomotion, and pro-depressant-like behavior [42].

Furthermore, three KOR agonists provided with different degrees of functional selectivity towards G protein-dependent signaling (i.e., BPHA, MCBPHA, and MCPPHA) were characterized concerning their neuroendocrine and behavioral effects: interestingly, KOR-dependent sedative effects elicited by these ligands were symmetrical to their ability to recruit arrestin 3 at KOR [43].

However, the G protein-biased KOR agonist RB-64 is surprisingly aversive in the conditioned place preference paradigm, although it does not induce anhedonia in other behavioral tests in rodents [44], while triazole 1.1 has been reported as non-dysphoric [45].

These findings highlight that not all the adverse effects elicited by classic KOR agonists distinctly depend on arrestin-mediated signaling. Thus, raising the need for further studies to understand better and corroborate the therapeutic potential of G protein-biased KOR agonist.

Overall, functionally selective agonists at MOR and KOR may still be considered candidates for potential opioid alternatives [46], and especially G protein-biased KOR agonists generally display an improved therapeutic window relative to unbiased agonists [47].

Nonetheless, considering that opioid-induced intracellular signaling is complex and multidimensional, as it is opioid-mediated modulation of therapeutic and adverse effects, efforts aimed at only reducing arrestin recruitment to opioid receptors might not be enough to improve the safety profile of opioids [32]. Thus, pointing out the need for a more comprehensive approach to identify, and account for, all the multifaceted mechanisms that may lead to an effective analgesic response devoid of the most relevant opioid-induced adverse effects.

## 3. Dawn and Dusk of Expectations on Biased Agonism at MOR: The Paradigmatic Course of Oliceridine (TRV130)

Oliceridine (also known as TRV130 and marketed under the name Olinvyk) is the first (and so far the only) G protein-biased MOR agonist that has been approved by FDA to treat moderate to severe acute pain in adults via short-term intravenous administration in hospitals or other controlled clinical settings. Despite its biased agonism, oliceridine displays a safety profile similar to other opioids: nausea, vomiting, dizziness, headache, and constipation are the most common adverse effects, with a boxed warning about addiction, abuse and misuse, and life-threatening respiratory depression. To our best knowledge, oliceridine has not been registered for use in Europe yet.

The course that led to oliceridine development and approval is highly paradigmatic regarding promises and pitfalls of G protein bias at MOR as an avenue for more effective and safer analgesics.

TRV130 was first reported by De Wire and coworkers as a MOR agonist preferentially activating G protein over arrestin in vitro, and eliciting antinociception with less severe gastrointestinal and respiratory adverse effects in rodents compared to morphine [48].

Consistent with this profile, in the following study on healthy volunteers, TRV130 produced analgesia with less reduction in respiratory drive and less severe nausea than morphine [49]. 

After that, in phase 2, randomized, placebo- and active-controlled study in acute pain following bunionectomy, it was shown that intravenous administration of TRV130 determined greater categorical pain relief as compared to morphine; however, no improvement in respiratory side effects was observed as compared to morphine [50].

In another phase 2, randomized clinical trial TRV130 produced analgesia in moderate-to-severe acute pain, suggesting efficacy with similar tolerability compared to conventional opioids [50]. Similarly, in a phase 2b study investigating effects on moderate to severe pain following abdominoplasty, oliceridine induced effective, rapid analgesia in patients with moderate to severe postoperative pain, with an acceptable safety/tolerability profile [51]. These findings demonstrate oliceridine efficacy in reducing moderate to severe postoperative pain in humans, although its therapeutic window became progressively narrower, moving from in vitro experiments to pre-clinical studies in animal models, healthy volunteers, and patients.

Interestingly, oliceridine retained undesirable constipating and abuse-related effects in rodents following repeated treatment, despite its bias for G-protein signaling [52]. Moreover, oliceridine was shown to elicit reinforcing and antinociceptive effects comparable to oxycodone in rats; thus, pointing out that a biased-signaling profile at MOR does not necessarily reduce abuse potential [53]. As a matter of fact, prevailing evidence suggests that G protein-biased MOR agonists, such as oliceridine, retain opioid-like abuse potential both in rodents and in humans [54]. These findings clearly show that the preferential activation of G protein- over arrestin-dependent signaling triggered by oliceridine may not be enough to achieve analgesia with milder adverse effects and reduced abuse liability, as compared to the pharmacological profile displayed by classic opioid agonists.

In October 2018, the US FDA committee voted against oliceridine approval as superior to morphine, although it was a tight vote (8 against, 7 in favor) [41].

This decision greatly dampened the enthusiasm and increased the skepticism around biased agonists targeting MOR as a promising strategy to develop improved analgesics.

Further studies highlighted that different G protein-biased MOR agents, such as oliceridine and PZM21, are partial agonists of MOR-mediated signaling to ion channels [55], thus, further fostering the increasing controversy on the actual molecular determinants of the improved therapeutic window displayed by these innovative MOR ligands.

Consistently with these observations, Gillis and coworkers proposed low intrinsic efficacy at G protein as a plausible alternative explanation for the improved pharmacological profile shown by ligands such as oliceridine and PZM21 [32].

Nonetheless, two further phase 3 clinical studies were carried out to assess oliceridine efficacy for treating moderate to severe acute pain following bunionectomy and abdominoplasty (i.e., APOLLO-1 and APOLLO-2 clinical trials) [56,57]. APOLLO-1 clinical study demonstrated that oliceridine is a novel and effective intravenous analgesic providing rapid analgesia for the relief of moderate-to-severe acute postoperative pain as compared to placebo [56], while the APOLLO-2 trial concluded that oliceridine is a safe and effective intravenous analgesic for the relief of moderate to severe acute postoperative pain in patients undergoing abdominoplasty [57]. These findings were further corroborated by the ATHENA clinical trial, a phase 3 open-label study showing that intravenous oliceridine is generally safe and well-tolerated for managing moderate to severe acute pain.

As a consequence of these and other results, FDA finally granted oliceridine approval in 2020, although as non-superior to classic opioids as regards its efficacy/safety profile.

To shed more light on oliceridine respiratory behavior, a comparative risk evaluation study has been carried out by exploiting data from previous trials and analyzing them via utility functions. This approach showed a favorable oliceridine safety profile over morphine when considering analgesia and respiratory depression over the clinical concentration range [58]. Nonetheless, other authors pointed out that whether oliceridine is a G protein biased or a partial agonist (evidence is strong for the latter) is immaterial if it provides good analgesic efficacy with a favorable side-effect profile [59]. The same authors, therefore, concluded that G protein bias at MOR is debatable as a simple pharmacological descriptor, given that partial agonism may be sufficient [59].

The course of oliceridine development and characterization is highly paradigmatic of the high promises and great expectations raised by the possibility of exploiting biased agonism at MOR as an avenue for more effective and safer analgesics, and of the subsequent pitfalls and disillusion related to all the controversial data that strongly challenged the foundations of this hypothesis.

More importantly, the course of oliceridine development and characterization has also emphasized the great complexity of differential MOR-mediated signaling pathways and how these processes may modulate therapeutic and adverse effects of opioid analgesics in turn. Highlighting again the need for a more comprehensive approach to fully understand if, and to what extent, biased agonism at MOR may be exploited to develop more effective and safer analgesics.

## 4. Quantitative Systems Pharmacology (QSP): An Innovative Strategy to Develop Improved Therapeutics to Tackle Complex Diseases through Analysis and Computation of All the Related Multidimensional Determinants

Although the process of drug discovery and development is paramount for modern medicine, it is becoming increasingly challenging for several reasons. The success rates are low: finding drug candidates that are both effective and with little or no side effects and bringing them to the market is highly expensive both in terms of economic resources and time (it costs from $1.2 to $4 billion and requires up to 10 years) [60].

Furthermore, the scaling up from the in vitro biochemical and pharmacological studies to the large, costly patient efficacy and safety studies is often compromised by a lack of understanding of drug behavior at the whole system level. Indeed, drug effects rely on the complex network of interactions between molecular structures, signaling pathways, and epigenetic remodeling. The complexity of this scenario is profoundly incremented by the differences in genetic backgrounds, development and disease states, and patient lifestyle and history, which influence the therapeutic and toxic effects elicited by medications. Therefore, there is an urgent need for innovative approaches to drug development that could account for evaluating all the multidimensional determinants involved in the disease of interest and drugs’ efficacy/safety profile. 

The emerging discipline of Quantitative and Systems Pharmacology (QSP), which derives from integrating Systems Biology and Quantitative Pharmacology, is well poised to support this need. In the last decade, both the Food and Drug Administration (FDA) and the National Institute of Health (NIH) have recognized the value of modeling and simulation in increasing productivity in biomedical research and pharmaceutical R&D [61].

An extensive working definition of QSP was provided by Sorger et al. in the NIH white paper published in 2011, which results from two workshops with experts from academia, industry, and government, including the FDA. In particular, QSP was defined “as an approach to translational medicine that combines computational and experimental methods to elucidate, validate and apply new pharmacological concepts to the development and use of small molecule and biologic drugs” [62].

Computer-aided modeling and simulation represent a move away from the “one drug one-target one-pathway” paradigm to a more network-centric view of pharmacology. Drugs may affect not only the intended target but also many other processes at the cellular, organ, and organism level. Computational and mathematical models allow testing of numerous potential scenarios in silico to exclude those associated with a low probability of success, avoiding the tremendous costs of evaluating all of those failed scenarios in the real world [63].

Within the drug discovery process, the QSP approach offers cheap predictive solutions for drug pharmacokinetic (PK), pharmacodynamics (PD), and patient-to-patient variability in therapeutic and adverse responses that will provide new discernments into fundamental biology and new ways to intervene therapeutically in disease pathophysiology, while minimizing toxicity [64].

QSP is innovative in its interdisciplinary approach, which incorporates concepts, methods, and investigators from classic pharmacology and pathology, biochemistry and structural biology, molecular genetics and medicine, as well as bioinformatics and the “-omics” sciences [62].

QSP integrates other modeling approaches routinely used in pharmaceutical R&D for several applications [60].

Pharmacokinetics (PK) attempts to study how an organism affects a specific xenobiotic/chemical after administration. In particular, it aims to examine the time-course of drugs’ absorption, distribution, metabolism, and excretion (ADME; e.g., dose-concentration relationships). Pharmacodynamics (PD) focuses on studying the biochemical and physiologic effects of drugs within animals (including humans), microorganisms, or combinations of organisms (e.g., infection). PD places particular emphasis on dose-response relationships. 

These latter disciplines are combined into the Pharmacokinetic/pharmacodynamic (PK/PD) modeling approach, which describes the time course of effect intensity in response to administration of a drug dose (e.g., exposure-response relationships). 

Physiologically-based pharmacokinetic (PBPK) models explain fundamental processes determining drug disposition at the tissue/organ level, including permeability across tissue barriers, organ blood flow, population variability and disease factors affecting these processes. Thus, providing a more detailed understanding and consumable prediction of the pharmacokinetic behavior of a compound in the body. The parameters describing PBPK models can be obtained from in vitro experiments, thus reducing the need for in vivo experiments [65].

Physiologically based pharmacokinetic/pharmacodynamic (PBPK/PD) models aim to achieve a mechanistic representation of the drug in biological systems by combining drug information on the physiology and biology at the organism level. These models include different organs and tissues; therefore, it is possible to quantitively estimate the drug exposure not only in plasma but also at the site of action, which may be difficult or impossible to measure experimentally.

Developing QSP models requires a multidisciplinary team that includes experts in modeling engineering, biology, pre-clinical and clinical studies, data programming, statistics, information technology, and pharmacology.

Although standard workflows utilizing QSP continue to evolve, in the rigorous and stepwise process of QSP development it is possible to identify the following relevant steps [60]:

- Model scope. In the first step, the therapeutic field and objectives of the model are delineated. The multidisciplinary team above-defined provides the physiological pathway map representing all of the biological and pharmacological processes associated with the model’s scope [61].
- Model development. In the second step, relevant non-clinical and clinical data from multiple sources are collected, and the raw data are converted to a suitable format. They can be derived from literature, biological databases, directly from the results of experiments carried out by the team that develops the model or, most commonly, by a combination of all these sources. Modelers develop mathematical descriptions of the processes and compartments involved in the interplay between drugs and pathophysiology.
- Model qualification. In the third step, the multidisciplinary team collects appropriate clinical data in patient populations necessary for the model qualification and calibration at relevant scales of physiology and time.

Once developed, a QSP model should be regularly updated with internal and external research. The output is a platform that integrates datasets from diverse studies, contexts, and spatiotemporal scales into a mathematical and computational framework that reflects our knowledge of the system.

Since 2011, when the term was coined for the first time, the number of recent original QSP researches and their applications in different diseases has increased significantly [60,63]. Many areas of medicine have begun applying QSP-informed approaches in drug development, clinical strategies, characterization of side effects, and set up a virtual patient cohort to support more personalized design therapies. These include immune-oncology, nutritional and metabolic diseases, nervous and digestive system diseases, cardiovascular diseases, and infections [60]. Additionally, QSP uncovers innovative therapeutic paradigms for complex multi-factorial diseases such as Alzheimer’s and diabetes. Because these diseases involve multiple physiological processes and can affect multiple organs, QSP can provide an integrated insight into the pathology and the possible complex counter-intuitive results of therapeutic intervention [61], as QSP holds great promise in accelerating drug discovery and development. From target identification to approval, it is proposed to identify and validate targets and druggable networks, uncover drug-response biomarkers, design better drugs and drug combinations, select appropriate doses and dosage regimens, and identify those patients most likely to respond to new therapeutic agents and combinations. It will therefore become a core discipline in the frame of translational medicine. What is more, QSP can address the drug efficacy and toxicity and can be used to document a drug’s likelihood of approval. Given their mechanistic nature, QSP models can also be utilized to discern the normal physiological behavior of molecules and cells in non-disease conditions.

Interestingly, due to their ability to account for and compute multiple complex and multidimensional data and information at the same time, QSP approaches can pave the way to fully understand and quantitatively describe the multifaceted connections between target activation (e.g., receptor stimulation by an agonist) and the resulting effects (e.g., therapeutic vs. adverse). Thus, offering an intriguing strategy to translate new pharmacological concepts (e.g., biased agonism at GPCR) into innovative drugs with improved efficacy/safety profiles.

## 5. Quantitative Systems Pharmacology: An Intriguing Approach to Go beyond the Greater Complexity of Opioid Receptor Pharmacology and the Current Limitations Entailing the Development of G Protein Biased Improved Analgesics

Opioid receptors pharmacology is complex and multidimensional. The differential activation of G protein-dependent over arrestin-mediated signaling pathways may lead to distinct cellular responses in vitro and, at least to some extent, to specific effects and behaviors in vivo.

Nonetheless, the ultimate outcome resulting from GPCR activation by different agonists depends on the modulation of a much more complex network of effectors and events: multiple G proteins (e.g., G_i1_, G_i2_, G_i3_, G_z_), as well as arrestin (i.e., arrestin 2 and arrestin 3) isoforms, may interact with opioid receptors [66,67], thus expanding the array of intracellular signaling events and processes that may be differentially modulated (also in a tissue-specific and time-specific fashion).

Additionally, opioid-mediated modulation of the same classes of intracellular effectors (e.g., ERK1/2 or JNK) may occur through both G protein- and/or arrestin-mediated processes, thus further increasing the complexity of opioid receptor pharmacology: classic KOR agonists, as U50,488 and dynorphin B, were shown to induce a biphasic activation of ERK1/2 and JNK phosphorylation in both rodent and human cells, with a G protein-dependent early phase and an arrestin 3-mediated late phase [42,68,69]. On the other hand, MOR analgesics such as morphine and fentanyl triggered JNK phosphorylation in an arrestin-independent and dependent way, respectively [70]. 

On top of that, it should be considered that such biphasic modulation of MAPK activation may contribute to the fine-tuning of other second messengers and mediators, whose balance is important in the frame of relevant cellular responses: it has been shown that arrestin-independent JNK activation by KOR agonists significantly increased ROS levels and that the arrestin-dependent JNK activation suppressed this ROS response [69]; providing some molecular determinants for the important implications of KOR agonists on modulating relevant physiological responses (e.g., stress responses) in a balanced way.

Furthermore, opioid receptors (as many GPCRs) are not isolated monads but may interact to form homodimers and heterodimers [71], providing an additional level of complexity to the pharmacological modulation of opioid receptors.

Moreover, physiological conditions (e.g., gender) and different signaling mechanisms in vivo may also deeply impact the effects elicited by biased agonists at opioid receptors. KOR-mediated, G protein-dependent antinociception is inactivated in female rodents by estrogen regulation of GRK2, while aversive effects induced by KOR agonists are observed in both males and females [72]. Accordingly, a G protein-biased KOR agonist is likely to determine no analgesia in females; this may be extremely relevant considering that chronic pain disorders are more prevalent in females than males.

Finally, chronic pain per se may promote relevant alteration at the systems level in terms of receptor and effector expression and function [73,74].

All these observations highlight that a comprehensive and multidimensional approach is required to effectively translate G protein bias at opioid receptors into innovative therapeutics with a truly improved pharmacological profile.

**Figure 1:**
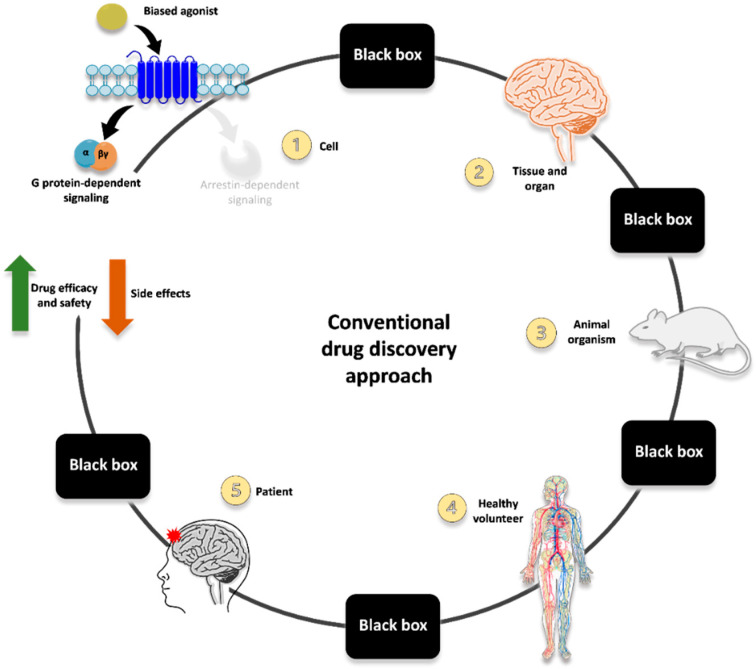
Schematic representation of the conventional approach to developing G protein-biased opioid agonists. One of the main challenges in contemporary medicinal chemistry is the development of safer and more effective analgesics for pain treatment. In the conventional drug discovery approach, the first goal for the development of biased ligands is the identification of agonists exerting their effects through functionally selective mechanisms and the association of these mechanisms with a desired in vitro cellular response (**1**). While G proteins and arrestins are undoubtedly critical signaling effectors that regulate both normal and abnormal physiology, attributing the complex GPCR signaling to proximal transducers is too reductive. Frequently, the signaling pathways directly responsible for the therapeutic and detrimental side effects are largely considered a black box because they are not thoroughly predictable and understandable. This is due to a lack of knowledge at the whole system level, which integrates multiple effectors and interdependent networks. Furthermore, the drug behavior is influenced by its exposure at the site of action in tissue/organ (**2**), which may be difficult or impossible to measure experimentally. Due to the many differences in physiological systems upon measuring bias, the translation from in vitro profiles of biased signaling into in vivo animal models (**3**) represents the major complication in the search for safer bias. The complexity of this scenario is profoundly incremented by the variability in drug response at the healthy volunteer (**4**) and patient (**5**) levels, which arises from differences in the proteome, genome, disease states, lifestyle, and history.

The route mainly employed until now for identifying G protein-biased opioid agonists is represented in Figure 1 and exemplified by the course of oliceridine development. 

Innovative ligands are typically screened for their ability to induce G protein activation and arrestin recruitment at the receptor (often employing one assay type per each of the two readouts; thus, not including multiple levels of possible functional selectivity and interplay between different signaling pathways). Then, hallmarks of G protein-dependent or arrestin-mediated signaling are evaluated in in vitro/ex vivo tissue preparation to corroborate the functional selectivity of the ligands (e.g., differential MAPK activation and production of second messengers and mediators). After that, the most promising candidate is evaluated in pre-clinical models recapitulating some behaviors/responses related to therapeutic and adverse effects (e.g., antinociception vs. constipation, respiratory depression, sedation). Once the in vitro and pre-clinical evidence is consistent and robust enough, studies can be carried out in humans, first in healthy subjects and then in specific patient categories.

Despite the promising therapeutic potential of functional selectivity, however, translating G protein-biased opioid agonists into novel therapeutics with minimized side effects is still far from being fully achieved. Furthermore, it is important to be reminded that recent evidence has accumulated challenging both the basic science foundation and translational application of biased agonists for improved pain therapy [19].

It is becoming increasingly clear that further studies are strongly needed to fully unravel the complexity of opioid receptor pharmacology so that all the different pathways and effectors potentially modulated are accounted for, and their connection with specific effects in vivo is fully characterized. It seems over-simplistic to reduce the multifaceted interplay between signaling pathways and the related therapeutic or adverse responses to the mere dichotomy between G protein activation and arrestin recruitment. Let alone the translation of in vitro G protein bias to in vivo pre-clinical and clinical outcomes (these latter being further complicated by patients’ proteome, genome, disease states, lifestyle, and history). 

Within this scenario, Quantitative Systems Pharmacology may provide an intriguing approach to extending beyond the greater complexity of opioid receptor pharmacology and the current limitations entailing the development of improved opioid analgesics. Employing multidimensional assays associated with computational tools to analyze large datasets has the real possibility of uncovering/identifying those signaling events and molecular pathways better correlated or directly connected to the desired phenotype of interest [75].

QSP platforms have already been successfully used in R&D processes devoted to developing innovative drugs for complex diseases (e.g., Alzheimer’s Disease) [60,76]. About fifty QSP models were published between 2019 and 2021, with cancer emerging as the most prominent application field, followed by metabolic diseases, nervous system diseases, and infections [60]. Recently, new research projects, such as QSPainRelief (a five years-long Horizon 2020 funded project started on January 2020, https://qspainrelief.eu/, accessed on 25 March 2022), undertook the challenging task of identifying new combination treatments for chronic pain through the development of QSP-based predictive platforms, further supporting the exploitation of Quantitative Systems Pharmacology to model complex and multidimensional events in the frame of developing improved therapeutics to treat chronic pain.

QSPainRelief main aim is to identify, assess and predict alternative combinations of existing opioid and non-opioid drugs with improved analgesia and reduced adverse effects using a mechanism-based QSP in silico model. 

This QSP platform is being implemented by integrating: a recently developed physiologically based pharmacokinetic model (PB/PK) to predict drug pharmacokinetics in human CNS; experimental in vitro data on pain/analgesia-related receptors/effectors expression and activation; experimental in vivo data on analgesic vs. adverse effects in pre-clinical and clinical studies; molecular modeling and dynamics of opioid receptor heteromers; a proprietary neural circuit model to predict drug effects on the activity of relevant brain neuronal networks. Such a platform also aims to integrate the newly produced results with existing clinical and real-world data; this to account as best as possible for relevant patient characteristics such as age, sex, disease status, and genotype.

This kind of QSP-based approach has a strong potential to identify new combinations of existing analgesics for improved treatment of chronic pain patients, providing at the same time relevant mechanistic information on the molecular determinants of chronic pain and analgesic/adverse effects as well as on the influence of age, sex, disease, and genetic factors.

**Figure 2:**
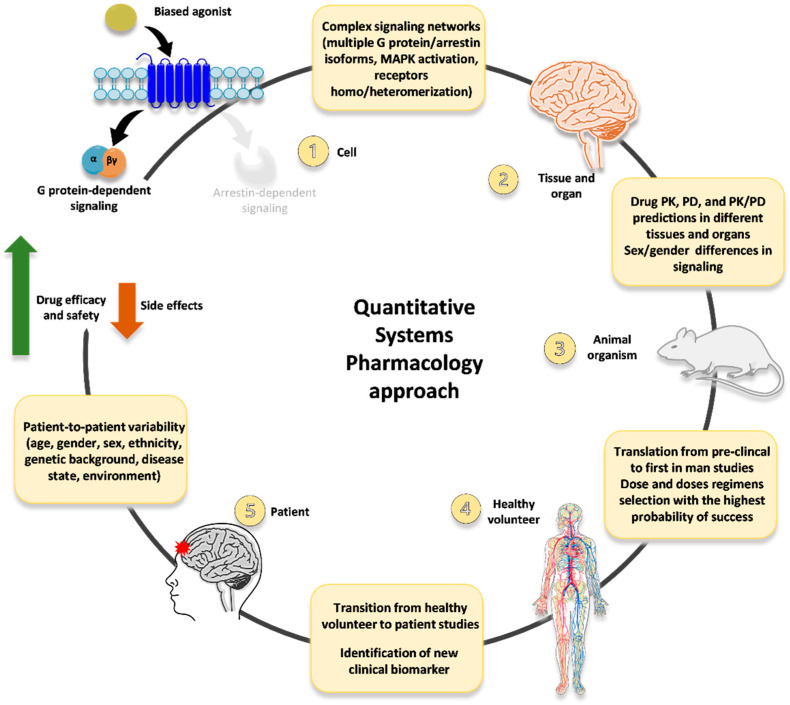
An innovative QSP-based approach for G protein-biased opioid agonists development. In both conventional and Quantitative Systems Pharmacology (QSP)-driven approaches, the key milestones for developing G protein-biased opioid agonists with improved efficacy/safety profiles are similar. However, mathematical modeling and sophisticated computation add quantitative and integrative perspectives (summarized in the yellow boxes) to activities that are currently qualitative or isolated. First, opioid receptor signaling is multidimensional and involves peculiar transducers/effectors. The multiple G proteins and arrestin isoforms, the biphasic modulation of MAPK (e.g., ERK1/2-JNK) through G protein- and/or arrestin-mediated processes, and the opioid receptors homo/heteromerization need to be included in this multifaceted scenario (**1**). QSP models focus on interactions among these multiple elements and may help uncover key and novel GPCR events related to the desired phenotype of interest and the disease pathophysiology. The creation of a multi-scale model that incorporates data at several temporal and spatial scales (biomolecules, cells, tissue, organ, organism) can provide more accurate predictions of bias ligand PK/PD relationships in tissues and organs and the probable outcome due to genre signaling differences (**2**), which deeply impact on the biased agonists’ effects. QSP can effectively promote the scale-up from animals to humans while correctly accounting for physiological and genetic differences. In particular, the platform may help to identify the dose and doses regimens with the highest probability of success (**3**), optimizing the clinical trial design and minimizing time and costs necessary for R&D. Furthermore, it may facilitate the transition from healthy volunteer to patient studies and the identification of new clinical biomarkers related to a specific response/behavior (e.g., analgesia, sedation, respiratory depression, cognitive impairment, abuse liability) (**4**). Finally, adopting a QSP approach to biased agonism at opioid receptors, which also incorporates patient-to-patient variability (**5**), may easily increase the likelihood of developing a successful G protein-biased opioid analgesic with increased efficacy/safety and lower side effects.

Moving from these considerations, we advocate an innovative, QSP-based approach to develop novel opioid ligands (including, but not limited to, G protein-biased agonists) as innovative analgesics with improved efficacy/safety profiles (Figure 2). First, in vitro data on multiple signaling readouts and intracellular pathways related to functional selectivity (not just the mere dichotomy between G protein activation vs. arrestin recruitment) are collected and employed to implement a QSP platform (so that the complex and multidimensional network that can be modulated downstream of opioid receptors is accounted for). Then, multiple hallmarks of functional selectivity (e.g., differential MAPK activation and production of second messengers and mediators) are evaluated in in vitro/ex vivo tissue preparations, and the resulting data is included in the QSP platform.

This way, the huge amount of information accounting for the multifaceted pharmacology of opioid receptors may be used to refine the selection of innovative drug candidates to be further investigated in different in vivo models recapitulating multiple responses/behavior of interest (e.g., analgesia, sedation, respiratory depression, cognitive impairment, abuse liability). All the results obtained in the frame of this extensive pre-clinical characterization may be, in turn, inserted into the QSP platform. Leading to implement a bioinformatic tool able to account for and at the same time compute all the relevant data and information describing the multifaceted scenario of biased agonism at opioid receptors (including in vivo differential conditions such as sex and age).

Moreover, relevant information on PK and PD in different tissues and organs, as well as any influence of sex-dependent differences in signaling pathways and molecular processes, could be included.

Such a QSP-based platform could then be employed to further refine the identification and selection of G protein-biased candidate agonists with the strongest possibility of displaying improved pharmacological profile in healthy volunteers and chronic pain patients. Then all the data and information obtained in clinical studies may be inserted into the QSP platform as well; moreover, relevant data could be included at this level regarding features of distinct subsets of patients (e.g., females and the elderly) and patient-to-patient variability. Thus, generating a comprehensive bioinformatic tool that may be used to refine further the identification and selection of innovative analgesics with increased efficacy and reduced side effects.

## 6. Challenges and Limitations of Current QSP-Based Approaches

QSP-based approaches provide quantitative and mechanistic platforms describing the phenotypic interaction between drugs, biological networks, and disease conditions to predict optimal therapeutic response [60]. Albeit highly promising in the frame of developing novel drugs for complex and multidimensional diseases, the implementation of QSP platforms may also pose relevant challenges and display significant limitations.

Despite the expansion of omics technologies and the increasing production and relevance of omics data, the latter is rarely included in the framework of QSP platforms. Although integrating omics findings into QSP platforms may help fill important biological and clinical data gaps, this would significantly increase the amount of information to be combined during model construction.

Moreover, experimental data at the biological scale of interest is necessary to parameterize QSP models; platform confidence largely relies on these quantitative results that, however, are sometimes missing [60]. These gaps may be filled by carrying out specific sets of new experiments and by searching the literature for the datasets that more closely match the required information. For instance, in developing a QSP platform aimed at identifying innovative opioid analgesics, opioid receptor protein expression levels at the target sites (e.g., dorsal root ganglia, cortical neurons, striatal neurons) represent a crucial piece of information. This kind of data could be generated via specific experiments carried out in vitro/ex vivo and/or obtained from literature data. 

Quantitative data on opioid receptors protein levels, though, are difficult to obtain/retrieve (mainly due to the lack of reliable antibodies), therefore, mRNA expression levels are often employed instead. However, considering that the correspondence between mRNA and protein levels is not always linear, the above-described approach may impact, at least in part, QSP platform accuracy.

Furthermore, sufficient quantitative mechanistic understanding may not be available yet for many systems, further complicating the development of QSP models that adequately describe the complexity of the system of interest. Under these conditions, additional in vitro and in vivo experiments are required to generate data for QSP development and calibration [77].

Another relevant challenge is that QSP models mainly focus on cell populations, receptors, effectors, and processes more prominently related to the condition of interest. Despite the possibility of including data and information from multiple cell types, different pre-clinical models, and clinical settings, the framework of all biological interactions in the environment is not fully integrated [60]. 

QSP models indeed allow extrapolating knowledge to predict outcomes in scenarios that have not been tested experimentally, making them an important resource in experimental and clinical pharmacology [78] and drug development processes. However, these models are usually complicated to work with due to their size and inherent complexity [78]. Therefore, some applications of QSP platforms to simulate relevant clinical settings, predict therapeutic outcomes, and estimate pharmacological parameters may become demanding from a computational perspective [78]. Different strategies have been developed to simplify QSP models in clinical pharmacology [78]. However, if, on the one hand, these simplification approaches significantly reduce the computational burden of QSP platforms, on the other hand, they may introduce some degree of information loss as compared to the full model.

Furthermore, QSP modeling may also require a human intervention to curate biological networks and literature review manually [60]. Developing a QSP platform entails extensive efforts and manual work carried out by a multidisciplinary team that outlines the biological scope and identifies the related components and interactions to properly define the model structure required to satisfy the modeling goals [63]. 

The complex nature of the biological systems may complicate the definition of the model scheme, given the importance of including the most relevant biological framework while avoiding the description of molecular processes that are not informative within the condition/disease/scenario of interest [63].

Notably, experiments carried out in different conditions or with different protocols may lead to conflicting findings. On the other hand, sparse and disparate (if not contradictory) results have been reported regarding complex and multidimensional scenarios such as biased agonism at opioid receptors. Raising the need within the model scheme definition to parse these data and establish appropriate computational protocols for properly incorporating them into QSP platforms [63]. This process may introduce some arbitrary corrections to the predicting models, thus potentially influencing, at least in part, the predictions for the downstream in vivo components of the QSP platform (see Figure 2). Different mitigation strategies have been conceived to overcome the limitations described above. The one recently explored the most, which seems valuable within this frame, is combining machine learning techniques (ML) with QSP approaches [60]. Adding ML to QSP may indeed contribute to minimizing prediction uncertainties, reducing bias in manual curation, and allowing automated data [60].

Individual-level clinical data from the population of interest is important to represent the clinical phenotype of interest in the model, both at the pathophysiological and organ or functional levels. Clinical biomarkers are also highly valuable for linking mechanisms in the QSP model to the appropriate outcome that can reflect improvement induced by a given therapy [63]. However, one of the challenges the modeling community faces is the limited availability of well-annotated datasets [79]. For some diseases, such as rheumatoid arthritis, many large trials are available spanning diverse mechanisms of action and well-established clinical measures used across these studies that can be used for model calibration and qualification. Conversely, there are fewer trials for Alzheimer’s disease. Nonetheless, neuroscience has been identified as a key disease area that would benefit the most from QSP models [79]. The availability of clinical data does not per se preclude the development and use of QSP models, but it can influence how simulation results are interpreted [79].

## 7. Concluding Remarks

Adequate management of chronic pain is still a challenge nowadays. Nearly two-thirds of patients, in fact, experience insufficient pain relief and/or relevant adverse effects induced by analgesic drugs.

Therefore, the need for more effective pain killers with limited adverse effects and negligible abuse liability is still largely unmet, despite the extensive efforts made in the past decades to identify and develop improved analgesics.

Biased agonism at opioid receptors has emerged as a promising avenue for more effective and safer analgesics. Raising remarkable enthusiasm and prompting increasing research efforts in the quest for G protein-biased MOR and KOR agonists as the basis to develop improved opioids.

Notwithstanding, this intense endeavor failed to provide innovative opioid ligands with improved efficacy/safety profile; the course of oliceridine development is paradigmatic concerning promises and pitfalls of G protein bias at MOR as an avenue for more effective and safer analgesics. 

The failure to translate functional selectivity into improved therapeutics is indeed due to the complex multidimensional pharmacology of opioid receptors: the multifaceted network of signaling events and molecular processes underlying therapeutic and adverse effects induced by opioids is, in fact, more complex than the mere dichotomy between G protein and arrestin and requires more comprehensive, integrated, network-centric approaches to be fully dissected. 

Within this frame, Quantitative Systems Pharmacology may provide an intriguing approach to go beyond the greater complexity of opioid receptor pharmacology and the current issues entailing the development of improved opioid analgesics.

Current QSP models also display some limitations. Different challenges must still be faced to refine these platforms into reliable predictive tools for identifying and selecting innovative analgesics with increased efficacy and reduced side effects.

Nonetheless, innovative QSP-based approaches, such as that proposed in Figure 2, have a strong potential to significantly advance the quest for novel analgesics with more favorable pharmacological profiles, due to the integration at the systems level of all the multiple determinants contributing to the multifaceted network of events involved in chronic pain on one hand, and in analgesic and adverse effects of innovative drugs on the other.

Last but not the least, if more QSP models are developed within opioid research, an increasing body of data and information will be generated, significantly helping advance the field of applying QSP to developing improved opioid analgesics.

## Data Availability

Not applicable.
